# Legislative: The Undertaker’s Bill, and Other Bills

**Published:** 1897-04

**Authors:** 


LEGISLATIVE: THE UNDERTAKER'S BILL, AND OTHER BILLS.—The Texas legislature is still in session—April 1st. Among the many bills introduced, "regulating" things, there is one entitled "An act to establish a State board of embalming; to provide for the better protection of health and life; to prevent the spread of contagious diseases, and to regulate the practice of embalming, and the care of and disposition of the dead." This is an advance along correct lines, and keeps step with civilization. We much prefer cremation, however, in the interest of sanitation.

The bill creates a State board of embalming, to consist of five members, to be appointed by the State Health Officer; term of service two years; except that of those first appointed three shall serve only one year. The State Health Officer to designate the term of service of each appointee. The members of said board shall be practical embalmers, having had experience in the business, and in the care of and disposition of dead human bodies. The appointing officer shall remove any member for neglect of duty, incompetence, or improper conduct. First board to be appointed on or before June 1st, 1897.

The duties of this board are defined to be, 1st. to prescribe a standard of proficiency as to qualification and fitness of those engaged in embalming and the care and disposition of dead bodies; 2nd, to meet as often as necessary; 3rd, elect officers; 4th, adopt a seal; 5th, adopt rules and regulations and by-laws to "regulate the practice" of embalming.

From and after July 1, 1897, every person now engaged or desiring to engage in the practice of embalming and the care and disposition of dead bodies, shall make written application for license,—fee of $5 to accompany application,—shall be of good moral character, shall have a pretty thorough knowledge of the gross anatomy of the human body—details specified in bill; also a knowledge of sanitation and of the process of disinfection of bodies, apartments, clothing, bedding, etc., in case of death from contagious or infectious diseases. If credentials and examination are satisfactory, the board shall issue a license, which must be recorded in the office of the clerk of the county court, and displayed in place of business of the holder. License to be renewed annually; fee of $2 for each renewal. Effective on and after September 1, 1897. Penalty for violation; misdemeanor punishable by fine of not less than $50 nor more than $100 for each and every offense; all fines collected to go to the public school fund of the State.

The State Health Officer is authorized to "grant dispensations to persons for the care and dispositon of dead bodies, exempting them from the requirement of this act, in communities having less than one thousand inhabitants."

The above is an excellent bill, and ought to become a law. Should it pass, — the willingness of the legislators to grant itonly, emphasises and makes more remarkable their refusal to require license to *practice medicine.* If it be necessary to the public health to license undertakers, to embalm and otherwise dispose of dead bodies, and to fix a standard of knowledge of the business and other qualifications, surely it cannot be denied that the practice of medicine is as dangerous a privilege; it gives license, virtually, to take life;—and no doubt, by the reckless indiscriminate practice of medicine, many lives are sacrificed, and it is never known. The legislature, for some reason, seems not disposed to so regard it. The bill to regulate the practice of dentistry, passed; ditto, pharmacy, passed; ditto, embalming—likely to pass. They will regulate "any old thing" except that which most needs—not "regulating"—for that word is, in my opinion, one cause of our repeated failures,—but *restricting:* Every person engaging in medical practice ought to conform to a standard of knowledge and qualification fixed by the authority of the State. and should be licensed by the State, and that license should be renewed every year—made a source of revenue to the the State. * *

The above brings up, of course, a consideration of the bill now before both houses, the Association's bill to "Regulate the Practice of Medicine." The writer was informed by a senator, during a recent visit (March 19) to the Senate hall, when inquiry was made as to the present status of the bill, that it is "so loaded down with amendments that its fathers will not know it when it comes up." As to when it would "come up," our informant said no one could tell; that after a separate amendment had been tacked on to include each so-called "school of practice"—the hydropaths, the vitopaths, the Christian scientists, the hypnotists, the spiritual healers and the "yerb doctor"—the friends of the bill had further consideration of it indefinitely postponed, subject to call.

The same old tactics, originating with our new found friends, the quacks; *ridicule.* It is most astonishing. There must be a cause for this deep-seated prejudice; this indifference to the effects, dangers and risks of the reckless, indiscriminate abuse of one of the most delicate and responsible trusts. At this writing the bill was about where it was at our last report—hanging fire in both houses.

